# 3D Target Localization of Modified 3D MUSIC for a Triple-Channel K-Band Radar

**DOI:** 10.3390/s18051634

**Published:** 2018-05-20

**Authors:** Ying-Chun Li, Byunggil Choi, Jong-Wha Chong, Daegun Oh

**Affiliations:** 1 Department Of Electronic Engineering, Hanyang University, Seoul 04763, Korea; davis.y.lee@hotmail.com (Y.-C.L.); jchong@hanyang.ac.kr (J.-W.C.); 2 Collaborative Robots Research Center, Daegu Gyeongbuk Institute of Science and Technology, Daegu 42988, Korea; choibk@dgist.ac.kr

**Keywords:** 3D localization, MUSIC, 3D shift invariant, 3D estimation, triple channel

## Abstract

In this paper, a modified 3D multiple signal classification (MUSIC) algorithm is proposed for joint estimation of range, azimuth, and elevation angles of K-band radar with a small 2 × 2 horn antenna array. Three channels of the 2 × 2 horn antenna array are utilized as receiving channels, and the other one is a transmitting antenna. The proposed modified 3D MUSIC is designed to make use of a stacked autocorrelation matrix, whose element matrices are related to each other in the spatial domain. An augmented 2D steering vector based on the stacked autocorrelation matrix is proposed for the modified 3D MUSIC, instead of the conventional 3D steering vector. The effectiveness of the proposed modified 3D MUSIC is verified through implementation with a K-band frequency-modulated continuous-wave (FMCW) radar with the 2 × 2 horn antenna array through a variety of experiments in a chamber.

## 1. Introduction

In recent years, much research has been done on the topic of localization by estimating the directions-of-arrival (DOA) (i.e., azimuth angles and elevation angles) and ranges of multiple targets in many applications, such as radar, sonar, and wireless communications [1,2,3]. Many methods based on one-dimensional (1D) uniform linear array (ULA) have been proposed for estimating azimuth angle [4,5,6,7], or joint range and azimuth angle estimation [8,9], such as the well-known multiple signal classification (MUSIC) algorithm [4] and estimation of signal parameters via rotational invariance techniques (ESPRIT) algorithm [5], and the generalized MUSIC and ESPRIT [6,7]. Two-dimensional (2D) DOA estimation for azimuth and elevation angle [10,11,12] has been further extended from the conventional 1D estimation methods based on some 2D arrays. Among the conventional works [8,9,10,11,12] on 2D estimation, in [9], a frequency-modulated continuous-wave (FMCW) radar system was implemented with the simplest form of ULA (only two receiving channels), and a dual smoothing algorithm was proposed for joint range and azimuth angle estimation. Obviously, the 1D structure of the two receiving channels of the radar system implemented in [9] restricts its ability to estimate the three-dimensional (3D) localization of targets, because the 1D structure of the two receiving array is not able to get two-dimensional angle information, azimuth, and elevation angle. However, 3D localization in a realistic environment is of greater interest, and will lead to further development of localization technology. To the best of our knowledge, there has been no research on the 3D MUSIC method implemented in conjunction with a K-band FMCW radar system. In [8], a 2D MUSIC algorithm for joint range and azimuth angle estimation was developed for an FMCW radar system, and its performance was analyzed by using simulated radar data. In [10], an ESPRIT-like method was proposed for 2D DOA estimation of coherent signals with a rectangular array, only verified by simulation results. In [11], 2D DOAs were estimated by using the 2D DFT-ESPRIT algorithms with a cylindrical conformal array. In [12], a recursive procedure based on an extended Kalman filter was proposed for 2D DOA estimation with a 3D antenna array, composed of three ULAs. Although the effectiveness of the suggested algorithms [8,10,11,12] has been demonstrated through simulations, they have not been verified by implementation in a system and corresponding experiments. However, in this work, the proposed modified 3D MUSIC method was implemented with a 2 × 2 horn antenna array, and its feasibility was verified through experiments using the implemented K-band radar system.

In this paper, we extend the antenna array structure from 1D in [9] to 2D to perform the joint 3D estimation of range, azimuth angle, and elevation angle. The implemented 2D antenna array structure has the simplest geometry of the planar array, and it is constructed as one miniaturized 2 × 2 horn antenna array composed of 4 small horn antennas, one as a transmitting antenna and the other three as receiving antennas. At the same time, the design of one K-band FMCW radar equipped with the constructed 2 × 2 horn antenna array is presented.

The conventional 3D MUSIC algorithm requires a 3D steering vector to calculate the 3D pseudo-spectrum, and the 3D steering vector is constructed by the Kronecker product of three 1D steering vectors. In our modified 3D MUSIC algorithm, an augmented 2D steering vector is proposed for the 3D MUSIC spectrum calculation. The proposed augmented 2D steering vector is obtained by connecting two 2D steering vectors in a specific way, which is related to how the stacked autocorrelation matrix is composed. The augmented 2D steering vector leads to more efficient computational complexity in 3D pseudo-spectrum calculation than the conventional 3D steering vector.

Several experiments were conducted in a chamber, and the results verified that the proposed modified 3D MUSIC algorithm implemented with the radar system with the 2 × 2 horn antenna array achieves good performance.

## 2. System Model

**Figure 1:**
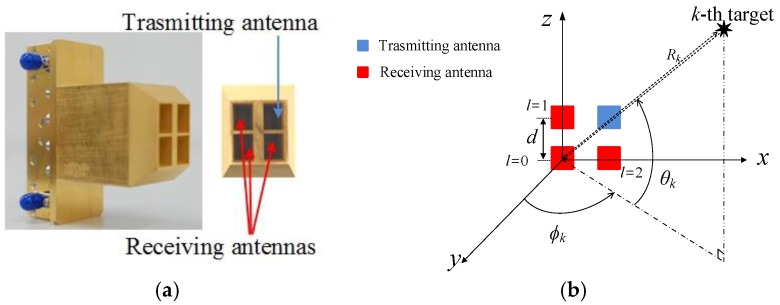
(**a**) Implemented 2 × 2 planar array with horn antennas; (**b**) Illustration of array configuration for joint range, azimuth, and elevation angle estimation.

As depicted in Figure 1a, we implemented a 2 × 2 horn antenna array, the array element at the upper-right corner is the transmitting antenna, and the other three elements are a triple-channel receiving antenna array. We consider the 2 × 2 horn antenna array at the *x–z* plane as shown in Figure 1b, and the receiving antenna elements at the origin, *z*-axis and *x*-axis are numbered as *l* = 0, 1, and 2, respectively. Thus, a ULA containing two elements is located on the *x*-axis, and the other ULA containing two elements is located on the *z*-axis. The array element spacing is *d* = *λ* (*λ* denotes the wavelength). More specifications of the implemented antenna will be introduced in Section 4.

In this paper, the triple-channel receiving antenna array processes narrowband plane waves incident on the sensor elements of the array. There are two types of system models: the vector wave model and the scalar wave model. In [13], the MUSIC method was handled with the vector wave model based on an electromagnetic field scattering model. In [8,9,14,15,16,17], the scalar models for the FMCW signals to estimate the range and angles have been suggested. Since our proposed method is developed with FMCW signals for joint estimation of range, azimuth, and elevation, the mathematical model in the Equations (1)–(6) come from the suggested mathematical model for FMCW radar signals in [8,9,14,15,16,17]. The relationship between the vector wave model and the scalar wave model was explained in [18] (the relationship between the Equations (7) and (20)).

We assume that the received signals from *K* targets impinge on the triple-channel receiving antenna array, including the information of {*ϕ_k_*, *θ_k_*, *τ_k_*}, *k* = 0, 1, …, *K* − 1, where *ϕ_k_*, *θ_k_*, and *τ_k_* are the azimuth angle, elevation angle, and time delay of the *k*-th target. We define *x_l_*(*t*) as the received signal of the *l*-th antenna element, and the signal representation of two ULAs along *x*-axis and *z*-axis can be extended from [17] as

(1) $$x\left(t\right)=\left[\begin{array}{c} x_{0}\left(t\right)\\ x_{1}\left(t\right)\\ x_{2}\left(t\right) \end{array}\right]=As\left(t\right)+n\left(t\right),$$

where

(2) $$\begin{array}{l} A=\left[a_{0}a_{1}\cdots a_{K-1}\right],\\ a_{k}=\left[\begin{array}{c} 1\\ p_{k}\\ q_{k} \end{array}\right],\\ p_{k}=\exp\left(-j\frac{2\pi d\cos\theta_{k}}{\lambda }\right),\\ q_{k}=\exp\left(-j\frac{2\pi d\cos\theta_{k}\sin\phi_{k}}{\lambda }\right),\\ s(t)=\left[\begin{array}{c} s\left(t-\tau_{0}\right)\\ s\left(t-\tau_{1}\right)\\ \vdots \\ s\left(t-\tau_{K-1}\right) \end{array}\right]. \end{array}$$

In the Equation (1), ***A*** denotes the array manifold matrix, ***s***(*t*) denotes the vector composed of the received signals of *K* targets, and ***n***(*t*) is the additive white Gaussian noise (AWGN) vectors whose elements have mean zero and variance. We define two electrical angles of the *k*-th target as *α_k_* = −2*πd*cos*θ_k_*/*λ* and *β_k_* = −2*πd*cos*θ_k_*sin*ϕ_k_*/*λ*, and one array factor vector of the *k*-th source ***a****_k_*, as shown in the Equation (2). Hence, we have the definition of array manifold matrix ***A*** as ***A*** = [***a***_0_ ***a***_1_ … ***a**_K_*_−1_]. In our implemented system, the components *s*(*t*) of received signal vector ***s***(*t*) are applied with the FMCW chirp signal, defined by

(3) $$s(t)=\{\begin{array}{c} \exp\left[j\left(f_{c}t+\frac{\mu }{2}t^{2}\right)\right]\;\mathrm{for}\;0\leq t<T_{sym}\\ 0\;\mathrm{elsewhere} \end{array},$$

where *f_c_* denotes the carrier frequency, *μ* is the rate of change of the instantaneous frequency of a chirp signal, and *T_sym_* is the duration of the FMCW chirp pulse. Based on the Equations (2) and (3), *x_l_*(*t*) in the Equation (1) can be rewritten as

(4) $$x_{l}\left(t\right)=\sum_{k=1}^{K}a_{k}\left(l\right)s\left(t-\tau_{k}\right)+n_{l}\left(t\right)\;\mathrm{for}\;l=0,1,2,$$

where ***a****_k_*(*l*) denotes the *l*-th elements of the vector ***a****_k_* in the Equation (2). The received signal in the Equation (4) will be moved to a beat frequency in a sinusoidal model by a mixer as

(5) $$y_{l}\left(t\right)=\sum_{k=1}^{K}a_{k}\left(l\right)y(t-\tau_{k})+{\tilde{n}}_{l}\left(t\right),$$

where

(6) $$y(t-\tau_{k})=\exp\left(j\left(\mu \tau_{k}t-\frac{\mu }{2}{\tau_{k}}^{2}+f_{c}\tau_{k}\right)\right),$$

and ${\tilde{n}}_{l}\left(t\right)$ denotes the transformed AWGN for the *l*-th antenna. Here, *y*(*t* − *τ_k_*) is the mathematical model for the beat signal, whose frequency is proportional to *τ_k_*, as in [14]. Thus, the signal model can be rewritten as

(7) $$Y=A\left[\begin{array}{c} y\left(t-\tau_{0}\right)\\ y\left(t-\tau_{1}\right)\\ \vdots \\ y\left(t-\tau_{K-1}\right) \end{array}\right]+\tilde{n}\left(t\right)=Ay\left(t\right)+\tilde{n}\left(t\right).$$

In (7), $\tilde{n}\left(t\right)$ denotes the transformed AWGN vectors composed of ${\tilde{n}}_{l}\left(t\right)$. The resultant beat signal in (7) will be converted with sampling frequency *f_s_* = 1/*T_s_* into sequences of samples *y_l_*[*n*] = *y_l_*(*nT_s_*) for *n* = 0, …, *N* − 1, where *N* = *T_sym_*/*T_s_*.

## 3. Proposed Algorithm

Since the proposed method is developed for joint estimation of the elevation angle, azimuth angle, and range for an FMCW radar with a triple-channel receiving array, we propose a stacked autocorrelation matrix, instead of a stacked Hankel matrix to exploit the 3D pseudo-spectrum estimation. Prior to explaining the spatially stacked autocorrelation matrix, the temporally averaged autocorrelation matrix and its factorization for one antenna is addressed with a mathematical model for noiseless data. Then, the matrix factorization is extended to the spatially stacked autocorrelation matrix. In the presence of noise, singular-value decomposition (SVD) of the stacked autocorrelation matrix and proposed 3D pseudo-spectrum estimation are developed.

### 3.1. Temporal Autocorrelation Matrix

The temporal autocorrelation matrix for the *l*-th element of the triple-channel receiving array can be defined based on the sampled sequences as in [19,20] by

(8) $$R_{l}=\sum_{n=0}^{N-L_{r}}y_{l,n}y_{l,n}^{H},$$

where ***y****_l_*_,*n*_ = [*y_l_*[*n*], *y_l_*[*n* + 1], …, *y_l_*[*n* + *L_r_* − 1]]*^T^*, [•]*^T^* denotes the transpose of a vector or a matrix contained within, and *L_r_* is the selection parameter that satisfies *L_r_* > *K*.

We provide the factorization model for the matrix ***R****_l_* with an assumption of no AWGN, and the transformation matrix ***T***_1_ such that

(9) $$R_{l}=PH_{l}QP^{H}T_{1},$$

where

(10) $$P=\left[\begin{array}{cccc} 1 & 1 & \cdots & 1\\ \zeta_{0} & \zeta_{1} & \cdots & \zeta_{K-1}\\ \vdots & \vdots & \cdots & \vdots \\ \zeta_{0}^{L_{r}-1} & \zeta_{1}^{L_{r}-1} & \cdots & \zeta_{K-1}^{L_{r}-1} \end{array}\right]\in {\mathbb{C}}^{L_{r}\times K},\;\mathrm{and}\;\zeta_{k}=\exp\left(j\mu \tau_{k}T_{s}\right),$$

(11) $$Q=diag\left[\rho_{0},\rho_{1},\ldots ,\rho_{K-1}\right]\;\mathrm{and}\;\rho_{k}=\exp\left(j\left(-\frac{\mu }{2}{\tau_{k}}^{2}+f_{c}\tau_{k}\right)\right),$$

(12) $$H_{l}=diag\left[a_{0}\left(l\right),a_{1}\left(l\right),\ldots ,a_{K-1}\left(l\right)\right].$$

Here, *diag*[•] denotes the diagonal matrix with the elements contained within the main diagonal.

### 3.2. Spatially Stacked Autocorrelation Matrix

A spatially stacked form of the autocorrelation matrix in the Equation (8) is expressed as

(13) $$R=\left[\begin{array}{l} R_{0}\\ R_{1}\\ R_{2} \end{array}\right]=\left[\begin{array}{l} PH_{0}QP^{H}T_{1}\\ PH_{1}QP^{H}T_{1}\\ PH_{2}QP^{H}T_{1} \end{array}\right]=AQP^{H}T_{1},$$

where

(14) $$A=\left[\begin{array}{l} {\mathrm{PH}}_{0}\\ {\mathrm{PH}}_{1}\\ {\mathrm{PH}}_{2} \end{array}\right]=\left[\begin{array}{cccc} 1 & 1 & \cdots & 1\\ \zeta_{0} & \zeta_{1} & \cdots & \zeta_{K-1}\\ \vdots & \vdots & \cdots & \vdots \\ \zeta_{0}^{L_{r}-1} & \zeta_{1}^{L_{r}-1} & \cdots & \zeta_{K-1}^{L_{r}-1}\\ p_{0} & p_{1} & \cdots & p_{K-1}\\ p_{0}\zeta_{0} & p_{1}\zeta_{1} & \cdots & p_{K-1}\zeta_{K-1}\\ \vdots & \vdots & \cdots & \vdots \\ p_{0}\zeta_{0}^{L_{r}-1} & p_{1}\zeta_{1}^{L_{r}-1} & \cdots & p_{K-1}\zeta_{K-1}^{L_{r}-1}\\ q_{0} & q_{1} & \cdots & q_{K-1}\\ q_{0}\zeta_{0} & q_{1}\zeta_{1} & \cdots & q_{K-1}\zeta_{K}\\ \vdots & \vdots & \cdots & \vdots \\ q_{0}\zeta_{0}^{L_{r}-1} & q_{1}\zeta_{1}^{L_{r}-1} & \cdots & q_{K-1}\zeta_{K-1}^{L_{r}-1} \end{array}\right].$$

**Figure 2:**
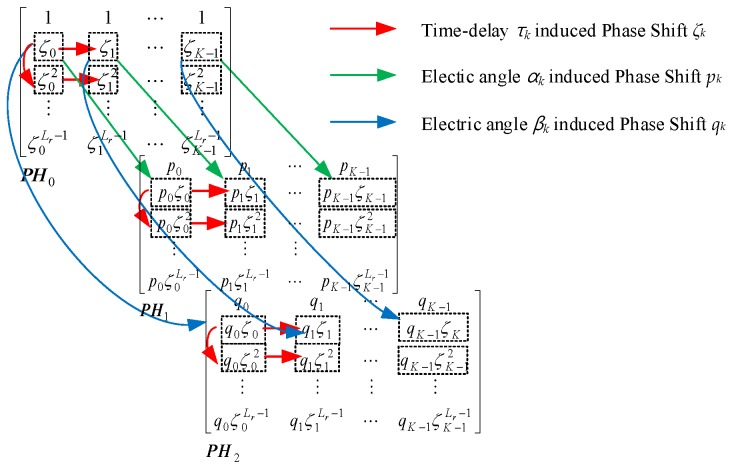
3D shift-invariant structure.

The phase shift *ζ_k_* in (10), *p_k_* and *q_k_* in (2), is composed of a time delay-induced element, an elevation-angle-induced element, and an elevation- and azimuth-angle-induced element, respectively. The 3D shift-invariant structure is shown in Figure 2.

### 3.3. SVD and Noise Subspace

In the presence of noise signals, the spatially stacked autocorrelation matrix ***R*** can be factorized like [21,22,23] in the subspace domain by SVD:

(15) $$R=U\Sigma V^{H},$$

where

(16) $$U=\left[\begin{array}{cc} U_{s} & U_{n} \end{array}\right],\Sigma =\left[\begin{array}{cc} \Sigma_{s} & \\ & \Sigma_{n} \end{array}\right]\;\mathrm{and}\;V=\left[\begin{array}{cc} V_{s} & V_{n} \end{array}\right].$$

Here, the submatrix ***U****_s_* = [***u***_1_ … ***u****_K_*] contains *K* eigenvectors that span the signal subspace of the matrix ***R***, and the submatrix ***U****_n_* = [***u****_K_*_+1_ … ***u****_Lr_*] contains *L_r_* − *K* eigenvectors spanning the noise subspace of the matrix ***R***. The values in the diagonal matrices ***Σ****_s_* = *diag*[*δ*_0_, *δ*_1_, …, *δ_K_*_−1_] represent the eigenvalues for the *K*-dimensional signal subspace, and values in the matrix ***Σ****_n_* = *diag*[*δ_K_*, *δ_K_*_+1_, …, *δ_Lr_*_−1_], *δ_K_* = *δ_K_*_+1_ = … = *δ_Lr_*_−1_ = $\sigma_{n}^{2}$, denote the noise variance, i.e., eigenvalues for the noise subspace of the matrix ***R***.

Since the signal subspace spanning matrix ***U****_s_* contains the first *K* eigenvectors of the matrix ***U*** as described previously, it requires exact knowledge of the number of signals *K*, in order to separate the signal and noise subspaces. The number of signals can be estimated by dealing with the singular values in a specific manner. The criterion of the minimum description length (MDL) [24,25] is adopted in this paper for classification of the signal and noise subspaces. We assume that the estimated signal subspace is correctly separated from the noise subspace, and the estimated number of targets is $\hat{K}$:

(17) $$\hat{K}=\underset{m\in \left\{0,1,\ldots ,L_{r}-1\right\}}{\arg\min}MDL\left(m\right),$$

where

$$\begin{array}{l} MDL\left(m\right)=-N\left(L_{r}-m\right)\log f\left(m\right)+\frac{1}{2}m\left(2L_{r}-m\right)\log N\\ \mathrm{and}\;f\left(k\right)={\left(\prod_{i=m}^{L_{r}-1}\delta_{i}\right)}^{\frac{1}{L_{r}-m}}\left(\frac{1}{L_{r}-m}\sum_{i=m}^{L_{r}-1}\delta_{i}\right). \end{array}$$

Since the number of signal $\hat{K}$ is obtained by the Equation (17), the signal subspace and noise subspace can be defined by

(18) $$R=\underset{\mathrm{signal}\;\mathrm{subspace}}{\underset{︸}{U_{s}S_{s}{V_{s}}^{H}}}+\underset{\mathrm{noise}\;\mathrm{subspace}}{\underset{︸}{U_{n}\Sigma_{n}V_{n}^{H}}}.$$

There can be the following relationship between the matrix ***A*** in the Equation (14) and the matrix ***U****_s_*, such that

(19) $$U_{s}=AT_{2},$$

where ***T***_2_ is a $\hat{K}$ by $\hat{K}$ non-singular transformation matrix, as in [26]. The matrix ***U***_s_ of the Equation (19) is the estimated signal subspace, and we assume the signal and noise subspaces are separated correctly. Since the steering matrix ***A*** of the Equation (14) shares the same signal subspace with the matrix ***U***_s_, the steering matrix ***A*** can be related to the estimated matrix ***U***_s_ based on the full rank transformation matrix ***T***_2_. If the signal and noise subspaces are separated incorrectly, the transformation between the matrix ***A*** and the matrix ***U***_s_ will not be available.

### 3.4. Modified 3D Steering Vector

Since the signal subspace and noise subspace are orthogonal, we propose a modified 3D MUSIC pseudo-spectrum estimation for the azimuth angle, elevation angle, and time delay. Assuming three pseudo-spectrum steering vectors, ***s****_z_* for time-delay estimation, ***s****_w_* for electrical angle *α_k_* estimation, ***s****_g_* for electrical angle *β_k_* estimation, are defined such that

(20) $$\begin{array}{l} s_{z}=\left[\begin{array}{cccc} 1 & \exp\left(j\frac{2\pi }{Z}z_{k}\right) & \cdots & \exp\left(j\frac{2\pi }{Z}\left(L_{z}-1\right)z_{k}\right) \end{array}\right]\in {\mathbb{C}}_{1\times L_{z}}\\ s_{w}=\left[\begin{array}{cc} 1 & \exp\left(j\frac{2\pi }{W}w_{k}\right) \end{array}\right]\in {\mathbb{C}}_{1\times 2}\\ s_{g}=\left[\begin{array}{cc} 1 & \exp\left(j\frac{2\pi }{G}g_{k}\right) \end{array}\right]\in {\mathbb{C}}_{1\times 2} \end{array}$$

respectively, where *z_k_* denotes the estimation for *ξ_k_*, *w_k_* denotes the estimation for *α_k_*, *g_k_* denotes the estimation for *β_k_*, and *L_z_* is the selection parameter. For the 2D MUSIC algorithms [27,28,29] for elevation angle and azimuth angle estimation or range and azimuth angle estimation, the Kronecker product ⊗ between the steering vectors for two electrical angles is organized as ***s****_w_*⊗***s****_g_*. While the Kronecker product is directly extended to the 3D steering vector ***s****_z_*⊗***s****_w_*⊗***s****_g_*, the stacked autocorrelation matrix must be defined as

(21) $$R=\left[\begin{array}{l} R_{0}\\ \begin{array}{c} R_{1}\\ R_{0} \end{array}\\ R_{2} \end{array}\right].$$

Comparing (21) to our definition in the Equation (13), ***R***_0_ is utilized only once, and a smaller matrix size is obtained in the Equation (13). Further, we propose an augmented 2D steering vector corresponding to the proposed stacked structure in the Equation (13). We first obtain two 2D steering vectors, by preforming the Kronecker product between ***s****_z_* and ***s****_w_*, ***s****_z_* and ***s****_g_*, respectively, such that

(22) $$s_{zw}=s_{z}⊗s_{w}\;\mathrm{and}\;s_{zg}=s_{z}⊗s_{g}.$$

In the Equation (22), we obtain two steering vectors ***s****_zw_* and ***s****_zg_*. As shown in Figure 1, the implemented triple channel receiving antenna array is composed of two ULAs, and the two ULAs share the same antenna element at the origin. Since the two ULAs will be used for elevation and azimuth angles respectively, the received signals from the antenna element at the origin are repeatedly used. Thus, the obtained two steering vectors ***s****_zw_* and ***s****_zg_* in the Equation (22) have the same elements, namely, the second half of ***s****_zw_* is same as the first half of ***s****_zg_*. Herein, we choose to delete the first half of ***s****_zg_*, and connect the ***s****_zw_* and the second half of ***s****_zg_*, and the augmented 2D steering vector for the modified 3D MUSIC algorithm can be obtained by

(23) $$s_{zwg}=\left[s_{zw}\left(\mathrm{the}\;\mathrm{second}\;\mathrm{half}\;\mathrm{of}\;s_{zg}\right)\right].$$

The 3D pseudo-spectrum can be obtained through the augmented 2D steering vector ***s****_zwg_* and the noise subspace spanning matrix ***U****_n_* in the Equation (16), such that

(24) $$Pseudo\left(z,w,g\right)=\frac{1}{s_{zwg}^{H}U_{n}U_{n}^{H}s_{zwg}}.$$

### 3.5. Transformation

Employing the peak detection method, the $\hat{K}$ peaks can be detected by 3D pseudo-spectrum searching, and the three estimated indexes ${\left\{z_{k},w_{k},g_{k}\right\}}_{k=0}^{\hat{K}-1}$ at which the $\hat{K}$ peaks are found, such that

(25) $$\left\{z_{k},w_{k},g_{k}\right\}={\left\{\max_{k}\left[Pseudo\left(z,w,g\right)\right]\right\}}_{k=0}^{\hat{K}-1},$$

where max*_k_*[•] denotes the *k*-th biggest value of the elements contained within. Note that the parameter matching for range, azimuth, and elevation sets is avoided. Since the three indexes of the 3D pseudo-spectrum are estimated, estimations for the elevation angle, azimuth angle, and time-delay of $\hat{K}$ targets, can be obtained by

(26) $${\hat{\theta }}_{k}=-\mathrm{acos}\left(\frac{\lambda }{\pi d}\times \frac{w_{k}}{W}\right),$$

(27) $${\hat{\phi }}_{k}=-\mathrm{asin}\left(\frac{\lambda }{\pi d\cos{\hat{\theta }}_{k}}\times \frac{g_{k}}{G}\right),$$

(28) $${\hat{\tau }}_{k}=\frac{c}{2\mu }\left(\frac{z_{k}}{Z}\times \frac{1}{T_{s}}\right),$$

respectively, where acos(•) denotes the inverse cosine function of the value contained within, and asin (•) denotes the inverse sine function of the value contained within.

### 3.6. Computational Burden Analysis

The costs for the required individual operations are summarized in Table 1. For the given data matrix ***R*** in Equation (13), the computational burden costs for the modified 3D MUSIC can be derived to be O(13$L_{r}^{3}$ + $L_{r}^{2}$*K* +*B*^3^*K*^3^$L_{r}^{3}$), where *B* is the iteration number for the three-dimensional searching. In general, the iteration number *B* is set to be much bigger than *M* and 13 for the high resolution 3D pseudo-spectrum. Hence, the derived computational complexity for the modified 3D MUSIC can be simplified to O(*B*^3^*K*^3^$L_{r}^{3}$). It implies that the computational burden is still expensive since the 3D spectrum searching is unavoidable.

## 4. System Implementation

In this section, implementation of the proposed K-band FMCW radar system with a small 2 × 2 horn antenna array is presented.

### 4.1. Transceiver and IF

**Figure 3:**
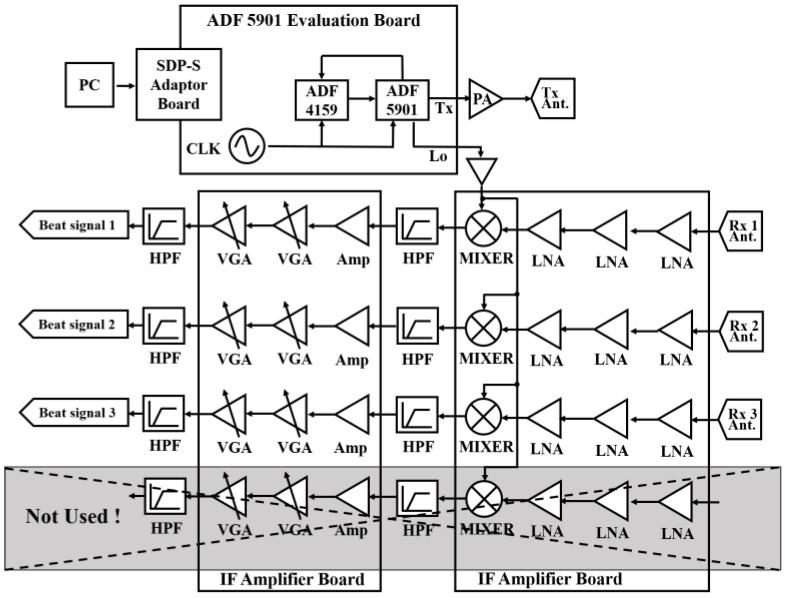
Block diagram of implemented triple-channel K-band radar system.

The proposed K-band FMCW radar system operates in the 24.025 to 24.225 GHz range with a 200 MHz bandwidth and a 100 μs period. The radar system comprises a 2 × 2 horn antenna array, an FMCW chirp generator ADF5901 evaluation module, a 4-channel receiver, an intermediate frequency (IF) amplifier, and a data logging platform, and so forth. A block diagram of the whole radar system is shown in Figure 3. The FMCW signals in the Equation (3) generated by the ADF5901 evaluation module are transmitted to the receiver as the local oscillator (LO) signal and to the transmitting antenna through a power amplifier (PA). The LO signals from the ADF5901 evaluation board are fed to the mixers of the triple receiving channels (one of the four channels is not used).

**Figure 4:**
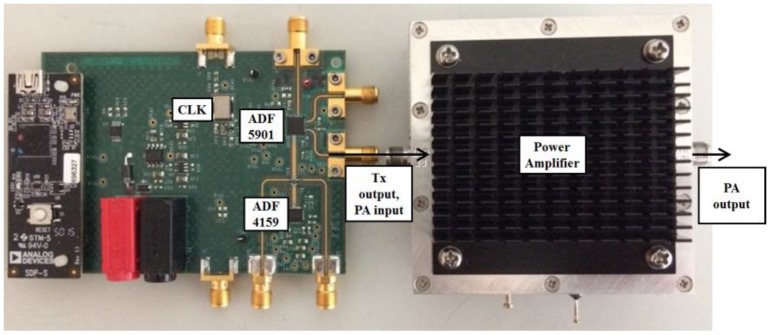
Frequency-modulated continuous-wave (FMCW) chirp generator ADF5901 evaluation board and 20 dB power amplifier.

As depicted in Figure 4, the ADF5901 evaluation board can provide the transmitted FMCW signal by the ADF5901 (voltage-controlled oscillator (VCO) and two output channels) in conjunction with the ADF4159 (fractional-N frequency synthesizer). Only one output channel of the ADF5901 evaluation board is used to feed the input of the PA for the transmitting antenna.

**Figure 5:**
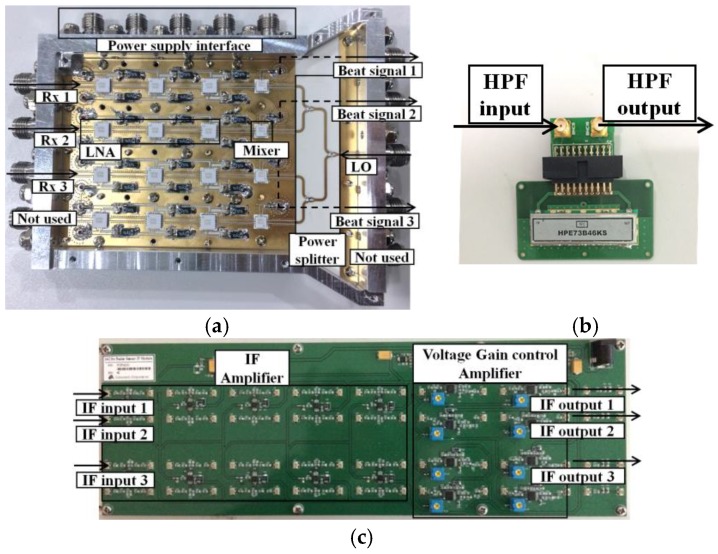
(**a**) Triple-channel receiver; (**b**) high-pass filter; (**c**) intermediate frequency (IF) amplifier.

The triple-channel receiver and IF amplifier are implemented as shown in Figure 5. Each receiving channel has three low noise amplifiers (LNAs) with −15 dBm P1dB output and one mixer, and the implemented receiving channel has a 10 dB maximum noise figure. The generated beat signals are amplified by the IF amplifier and voltage gain control (VGA) amplifier, and then the amplified IF signals are processed by the proposed algorithm. One high-pass filter (HPF) is utilized between the mixer and IF amplifier, and more specifications of the HPF will be explained in next subsection.

**Figure 6:**
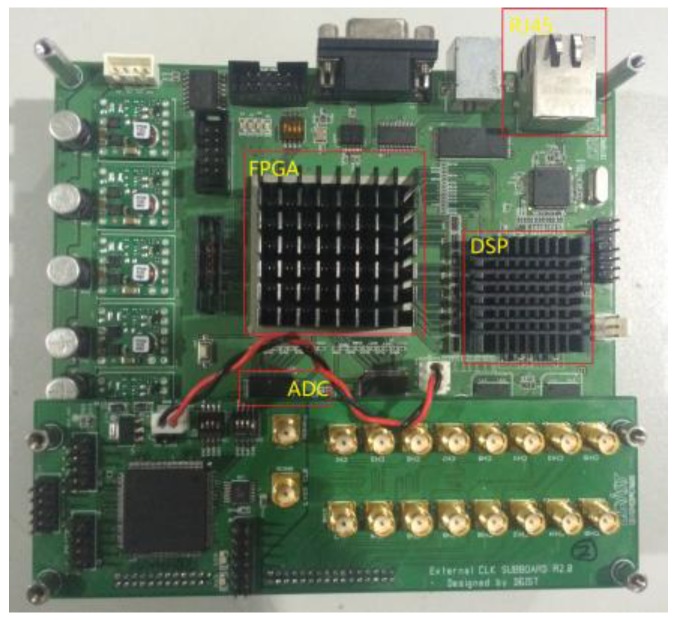
Data-logging platform.

The received beat signal was sampled through a field programmable gate array (FPGA) and a digital signal processing (DSP) board as shown in Figure 6. The analog to digital convertor (ADC) in the implemented radar system has a 12 bit output (72 dB dynamic range), and a 12.5 MHz sampling rate.

The parameters of the implemented K-band radar system are summarized in Table 2.

### 4.2. 2 × 2 Horn Antenna Array

**Figure 7:**
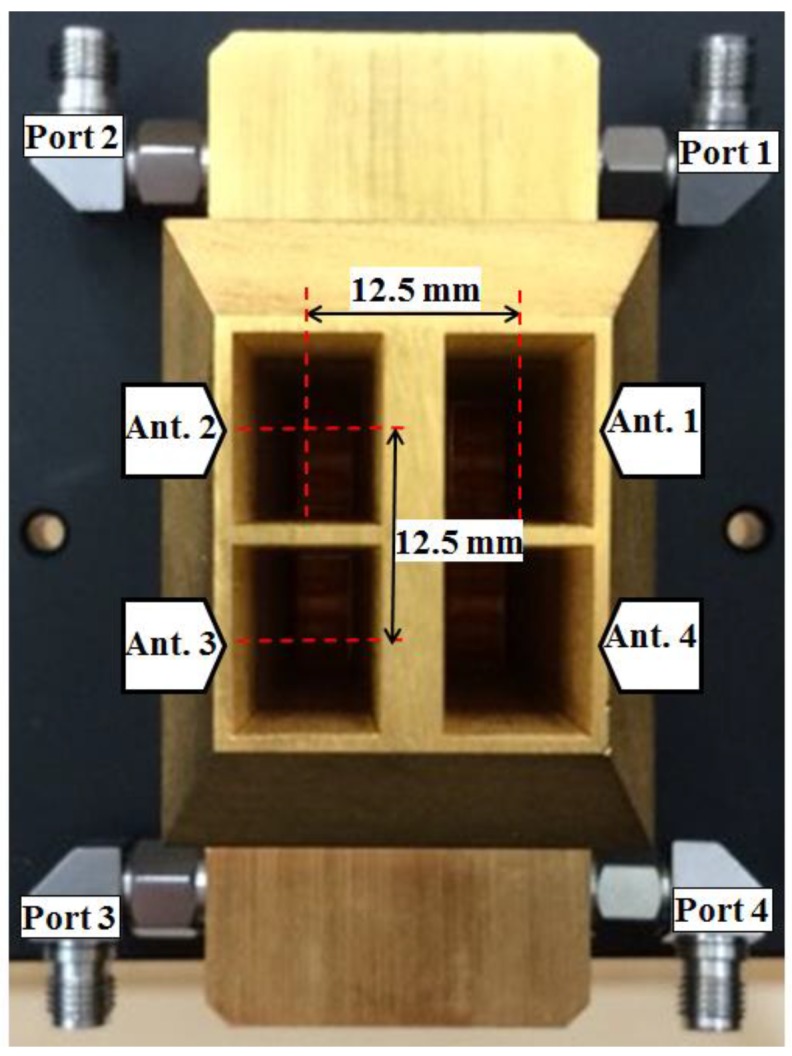
Transmitting antenna and receiving antennas.

**Figure 8:**
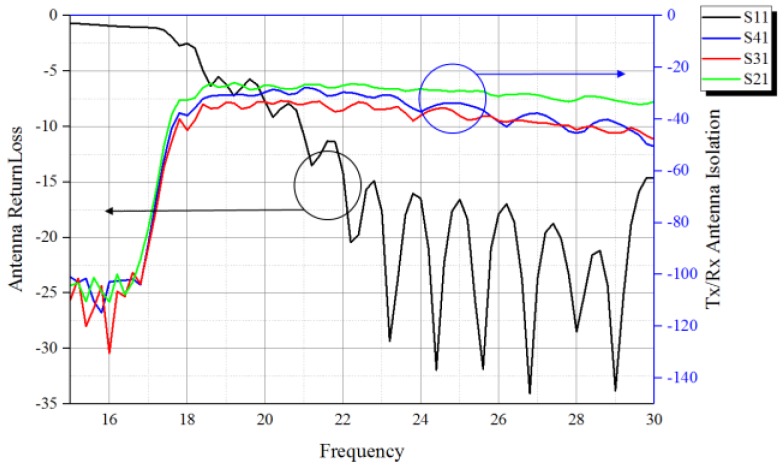
Transmitting antenna return loss and receiving antennas isolation characteristics.

**Figure 9:**
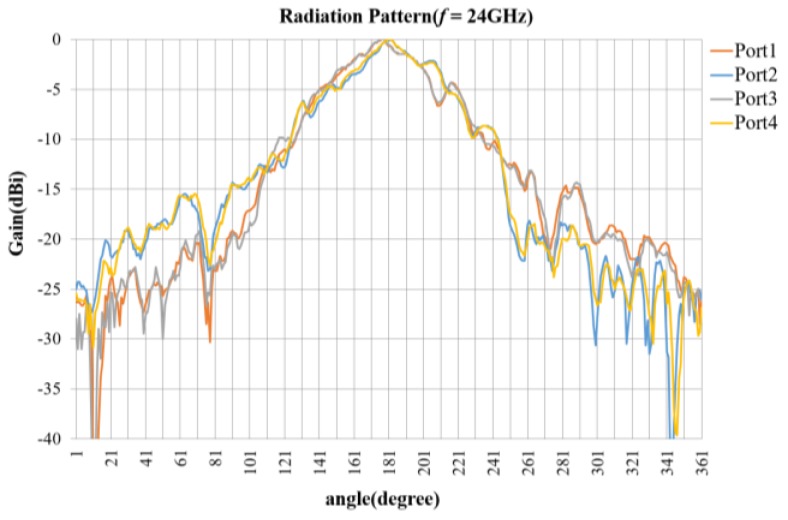
Radiation pattern of antenna elements.

The 2 × 2 horn antenna array is implemented as shown in Figure 7. As mentioned in Section 2, the array element (Ant. 1) at the upper-right corner is the transmitting antenna, and the other three elements (Ant. 2–4) are a triple-channel receiving antenna array. We identify the input ports as Port 1–4 for the antennas Ant. 1–4, respectively, to explain the characteristics as shown in Figure 8 and Figure 9.

Figure 8 shows the transmitting antenna return loss and adjacent receiving antenna isolation characteristics over the range of 21 GHz to 30 GHz. The performance was tested by a network analyzer. S11 means the return loss of the transmitting antenna Ant.1, and S21, S31, and S41 represents the power transferred from Port 1 to Port 2, Port 1 to Port 3, and Port 1 to Port 4, respectively. It should be noted that the isolations between the transmitting antenna and the receiving antenna array were measured as −28 dB (max.) at 24 GHz; therefore, the HPF, as shown in Figure 3 and Figure 5b, is used in the receiver to mitigate the isolation effectiveness.

Figure 9 shows the normalized radiation pattern of a single element of the implemented 2 × 2 horn antenna array, and each element has a similar pattern, which has 8 dBi antenna gain and 45° 3 dB beam width.

## 5. Experiments and Results

Experiments were conducted to test the performance of the proposed 3D MUSIC algorithm and the developed K-Band FMCW radar with the 2 × 2 horn antenna array. The received data of the radar system were sampled by an FPGA and DSP board, and then they were processed by the proposed algorithm in MATLAB 2016b. For each experiment, 100 repeated detection trials were conducted.

**Figure 10:**
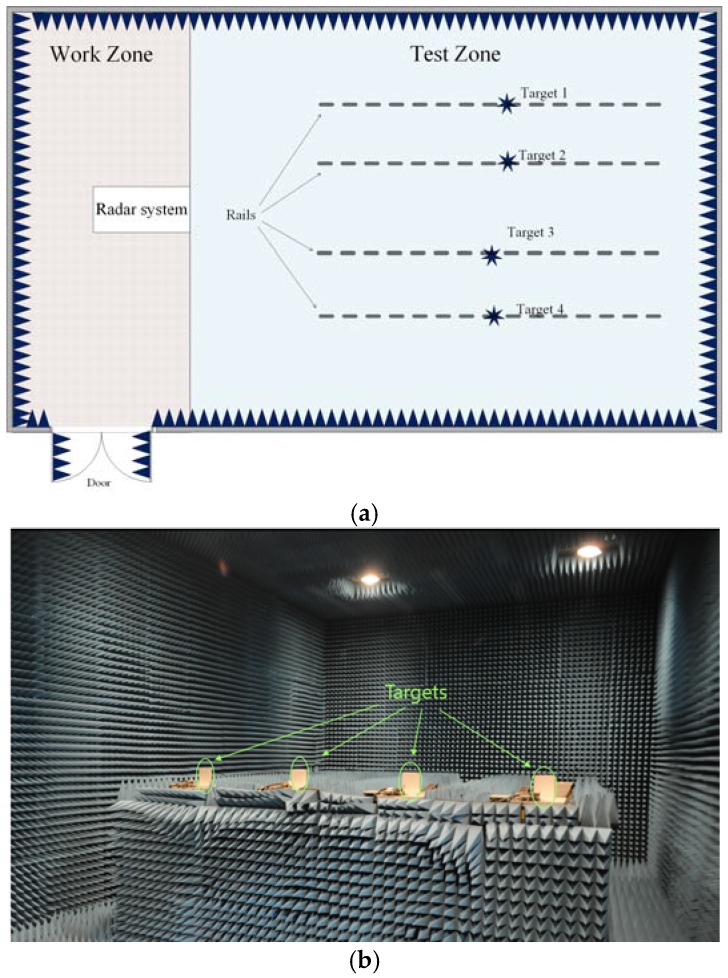
Experiment scenario in the chamber: (**a**) layout; (**b**) inside view.

The experimental set up of the chamber in Daegu Gyeongbuk Institute of Science & Technology (DGIST, Daegu, Korea) is shown in Figure 10. The implemented antenna array was set up on one pillar at the “Radar system” location of Figure 10, and the position of the Ant. 3 was assumed to be the origin of the Cartesian coordinates. Hence, the Cartesian coordinate system was implemented in the same way as shown in Figure 1, with the *z*-axis along the pillar and the *y*-axis along the boresight of the antenna array. There were four targets (four small iron blocks with 10 cm side length) in the same horizontal plane mounted on four different rails inside the test zone.

Four sets of experiments were conducted to detect the immobile targets in the chamber, respectively, and the locations of targets for the four sets of chamber experiments are shown as the form (*x*, *y*, *z*) in Table 3.

**Figure 11:**
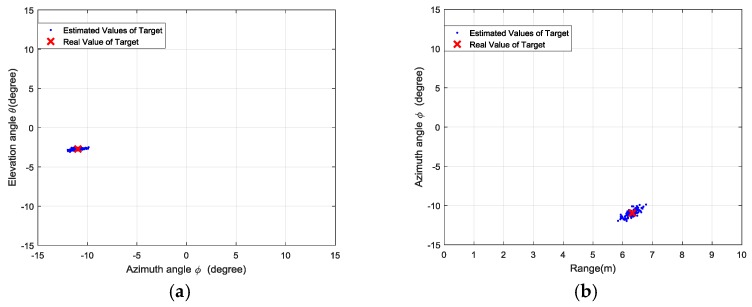

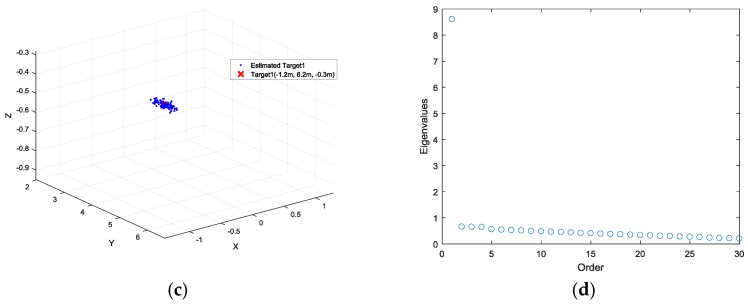
Results of the first set of experiments: (**a**) azimuth angle and elevation angle estimation; (**b**) range and azimuth angle estimation; (**c**) 3D view of target locations; (**d**) distribution of singular values.

**Figure 12:**
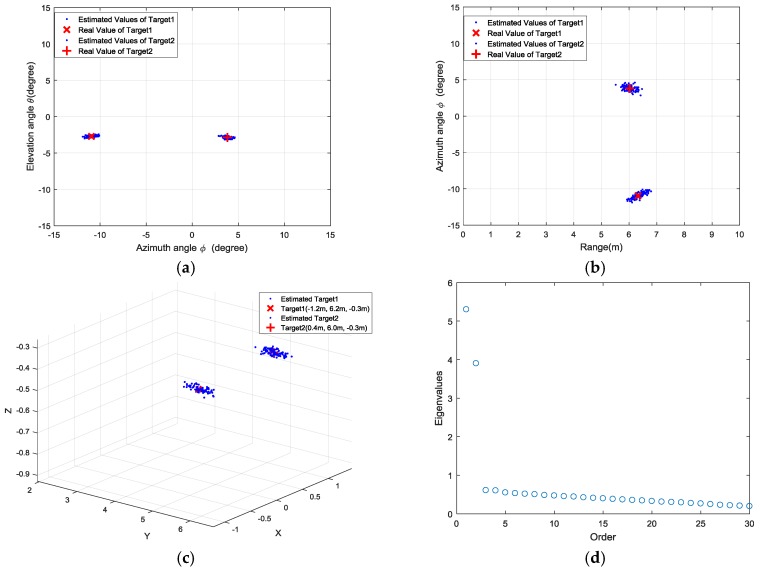
Results of the second set of experiments: (**a**) azimuth angle and elevation angle estimation; (**b**) range and azimuth angle estimation; (**c**) 3D view of target locations; (**d**) distribution of singular values.

**Figure 13:**
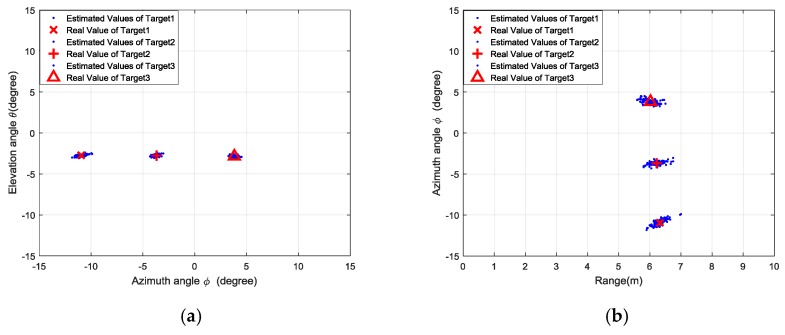

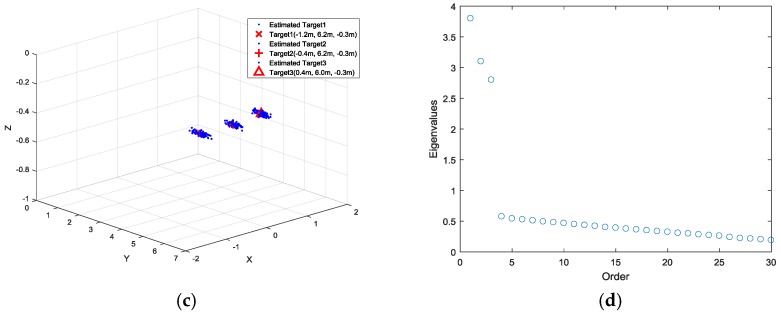
Results of the third set of experiments: (**a**) azimuth angle and elevation angle estimation; (**b**) range and azimuth angle estimation; (**c**) 3D view of target locations; (**d**) distribution of singular values.

**Figure 14:**
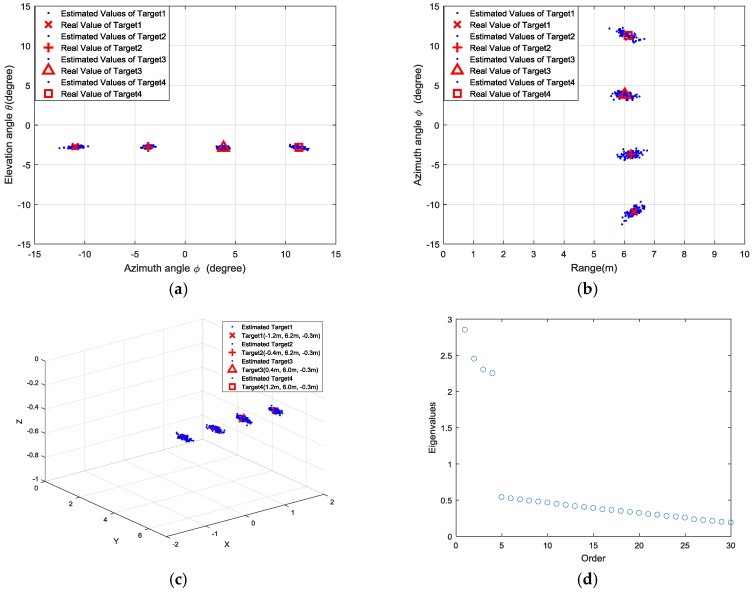
Results of fourth group experiments: (**a**) azimuth angle and elevation angle estimation; (**b**) range and azimuth angle estimation; (**c**) 3D view of target locations; (**d**) distribution of singular values.

We conducted 100 trials for each set of experiments, and the estimated locations are presented as the azimuth-elevation angle map and range-azimuth map in Figure 11, Figure 12, Figure 13 and Figure 14, respectively.

The root mean square error (RMSE) was selected as the measurement of the experiment results, and we defined the RMSE for each target as $\sqrt{\frac{1}{100}\sum_{b=1}^{100}{\left({\hat{x}}_{b}-x\right)}^{2}+{\left({\hat{y}}_{b}-y\right)}^{2}+{\left({\hat{z}}_{b}-z\right)}^{2}}$, where $\left({\hat{x}}_{b},{\hat{y}}_{b},{\hat{z}}_{b}\right)$ denotes the estimated position of the target (*x*, *y*, *z*) from the *b*-th trial. The measured RMSE values for all experiments are shown in Table 4.

## 6. Conclusions

This paper extended the previous work [9] to obtain the joint 3D estimation for range, azimuth, and elevation angle. An augmented 2D steering vector based on the proposed stacked autocorrelation matrix was proposed. The 3D pseudo-spectrum of the modified 3D MUSIC algorithm is constructed through the proposed augmented 2D steering vector. Since the augmented 2D steering vector has a smaller size than the conventional 3D steering vector, the 3D pseudo-spectrum calculation based on the augmented 2D steering vector obtains more efficient computational complexity than that based on the conventional 3D steering vector. At the same time, the modified 3D MUSIC algorithm avoids parameter matching for range, azimuth, and elevation sets. Several sets of chamber experiments were conducted to verify the performance of the proposed algorithm implemented with a K-band radar system with the 2 × 2 horn antenna array. The experimental results demonstrated the effectiveness of the proposed algorithm for 3D estimation of range, azimuth, and elevation angles. Although the proposed augmented 2D steering vector is utilized in the modified 3D MUSIC algorithm, the computational complexity is still high for real-time applications, and 3D spectrum searching is needed for the obtained 3D pseudo-spectrum.

## Figures and Tables

**Table 1:** Costs of individual operations.

Operation Description	Computational Complexity
SVD of ***R***	*O*((3*L_r_*)^2^*L_r_* + *3L_r_*$L_{r}^{2}$ + $L_{r}^{3}$) = *O*(13$L_{r}^{3}$)
***U*** *_s_*	*O*($L_{r}^{2}$)
Three-dimensional searching	*O*(*B*^3^*K*^3^$L_{c}^{3}$)

**Table 2:** Summary of system specifications.

Parameter	Specification
Modulation type	FMCW
Carrier frequency	24.025 GHz~24.225 GHz
Bandwidth	200 MHz
Sweep time	100 μs
*Tx* and *Rx* antenna	2 × 2 horn antenna array
Number of Rx channels	3 Channel
EIRP	28 dBm
Receiver noise figure	10 dB
Receiver RF maximum gain	50 dB (Max.)
Maximum IF gain	40 dB (Max.)
Receiver dynamic range	72 dB
RF power consumption	4 W

EIRP: equivalent isotropically radiated power.

**Table 3:** The locations of targets (unit: m).

	Target 1	Target 2	Target 3	Target 4
1st experiment	(−1.2, 6.2, −0.3)			
2nd experiment	(−1.2, 6.2, −0.3)	(0.4, 6, −0.3)		
3rd experiment	(−1.2, 6.2, −0.3)	(−0.4, 6.2, −0.3)	(0.4, 6, −0.3)	
4th experiment	(−1.2, 6.2, −0.3)	(−0.4, 6.2, −0.3)	(0.4, 6, −0.3)	(1.2, 6, −0.3)

**Table 4:** Measured RMSE values.

	Target 1	Target 2	Target 3	Target 4
1st experiment	0.1725			
2nd experiment	0.1773	0.1768		
3rd experiment	0.1806	0.1784	0.1788	
4th experiment	0.1832	0.1812	0.1795	0.1821
